# An Ultra‐Fast Rolling Double‐Helical Robot Driven by Constant Humidity

**DOI:** 10.1002/advs.202500577

**Published:** 2025-04-02

**Authors:** Chuhan Xu, Jiayao Ma, Lei Fu, Xinmeng Liu, Lei Zhang, Yan Chen

**Affiliations:** ^1^ Key Laboratory of Mechanism Theory and Equipment Design of Ministry of Education Tianjin University 135 Yaguan Road Tianjin 300350 China; ^2^ School of Mechanical Engineering Tianjin University 135 Yaguan Road Tianjin 300350 China; ^3^ Department of Biochemical Engineering Frontier Science Center for Synthetic Biology and Key Laboratory of Systems Bioengineering (MOE) School of Chemical Engineering and Technology Tianjin University Tianjin 300350 China; ^4^ Haihe Laboratory of Sustainable Chemical Transformations Tianjin 300192 China

**Keywords:** constant humidity, double‐helical structure, rolling locomotion, soft robot, stimuli‐responsive materials

## Abstract

Untethered soft robots made of stimuli‐responsive materials hold great application potential in various fields. However, most robots of this type require artificial modulation of the stimuli to actuate, while it is a great challenge to achieve fast periodic locomotion under a constant external environment. Here, a double‐helical robot constructed with humidity‐sensitive agarose (AG) films, referred to as the Dualicalbot is proposed, which can rapidly roll under a constant humid environment by making two helices alternately bend by absorbing humidity to actuate the robot in two half‐cycles. A theoretical model is built to unveil the periodic deformation of the robot as well as the correlation between the design parameters and the motion speed, based on which the Dualicalbot can reach a maximum rolling speed of 5.8 BL s^−1^. Moreover, it is capable of carrying a payload up to 100% of self‐weight and detecting the acid environment it rolls through. This work is envisaged, and more generally the structural design and theoretical modeling principle, will open a new avenue for the development of advanced soft robotics with diverse functionalities.

## Graphical abstract

A double‐helical robot named *Dualicalbot* is proposed to achieve a high‐speed rolling under a constant humidity. The special asymmetric structural design makes sure that the two helices alternately drive the robot to roll with a remarkable speed of 5.8 BL s^−1^, 4.8 times faster than the fastest robot driven by a constant environment and surpassing the majority of untethered soft robots driven by modulated stimuli.

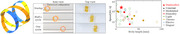

## 1 Introduction

Stimuli‐responsive materials, which are capable of undergoing large deformation or generating driving forces in response to a broad spectrum of stimuli, including electricity,^[^ ^1^, ^2^, ^3^, ^4^, ^5^, ^6^ ^]^ magnetic fields,^[^ ^7^, ^8^, ^9^, ^10^, ^11^, ^12^, ^13^, ^14^, ^15^, ^16^, ^17^, ^18^ ^]^ heat,^[^ ^19^, ^20^, ^21^, ^22^, ^23^, ^24^ ^]^ light,^[^ ^25^, ^26^, ^27^, ^28^, ^29^, ^30^ ^]^ humidity,^[^ ^31^, ^32^, ^33^, ^34^, ^35^, ^36^, ^37^ ^]^ and even multiple stimuli,^[^ ^38^, ^39^, ^40^, ^41^, ^42^, ^43^, ^44^ ^]^ have demonstrated great potential in the development of advanced soft robots with application scenarios including cargo transportation,^[^ ^19^, ^22^, ^34^, ^39^, ^41^ ^]^ medical treatment,^[^ ^9^, ^14^, ^17^, ^18^, ^44^ ^]^ sensing,^[^ ^45^, ^46^, ^47^ ^]^ and mapping,^[^ ^22^ ^]^ and so on.

Soft robots made from stimuli‐responsive materials typically require artificial modulation of the external stimuli, such as changing the direction of electrical or magnetic fields, light intensity or position, or temperature, so as to generate periodic deformation of the smart materials.^[^ ^48^ ^]^ The external control endows this type of soft robot with remarkable mobility and diverse motion modes.^[^ ^6^, ^7^, ^13^, ^14^, ^15^, ^16^, ^17^, ^18^, ^42^, ^43^, ^44^ ^]^ By combining sheet‐like structures with anisotropic friction feet, various crawling robots with multiple functions have been developed.^[^ ^6^, ^7^, ^11^, ^12^, ^13^, ^40^, ^43^ ^]^ Mao et al. utilized liquid metal channels embedded in a curved elastomeric bilayer to create an electromagnetic crawling robot capable of reaching speeds up to 70 body lengths per second (BL s^−1^) and multimodal motion through varying the actuation frequency.^[^ ^6^ ^]^ Beyond sheets, tubular structures, including cylinders and helicoids, are also employed to achieve rolling motion.^[^ ^27^, ^41^ ^]^ For instance, Choi et al. developed a center‐tapered helicoid robot fabricated from liquid crystal polymer networks with a maximum speed of 21.63 BL s^−1^ powered by a follower light source, and demonstrates its ability to steer and swim.^[^ ^30^ ^]^ However, in certain scenarios, their reliance on external guidance constrains their applicability due to a lack of autonomy.

To improve the autonomy of the soft robots, efforts have been put into designs capable of motion in a constant environment without artificial modulation.^[^ ^49^ ^]^ The key challenge here is how to generate self‐sustained period deformation in response to constant stimuli, to address which researchers resort to specially designed robot structures.^[^ ^50^ ^]^ By constructing asymmetric deformation, sheet‐like structures can achieve continuous rolling motion in a constant environment. Fu et al. attached two rotationally symmetric PET feet to both ends of a humidity‐sensitive agarose film to induce a periodic offset of the barycenter, and developed a rolling robot with a speed of 0.73 BL s^−1^.^[^ ^34^ ^]^ Zhou et al. altered the principal direction of strain in a single liquid crystal elastomer film, and realized a rolling locomotion at a maximum speed of 1 BL s^−1^. Moreover, tubular structures can also realize rolling through alternative deformation of different parts of the structure.^[^ ^24^ ^]^ Kotikian et al. designed a pentagonal prism robot, which could roll unidirectionally by having each edge in orderly contact with the heated substrate.^[^ ^23^ ^]^ Zhai et al. developed a helical robot fabricated by double layer 4D printing, which could realize untethered rolling on a heated surface.^[^ ^19^ ^]^ Nevertheless, the locomotion speeds of those robots are still relatively low in comparison with those actuated by modulated stimuli.

To tackle this challenge, here we propose a soft robot named *Dualicalbot* with the aim of achieving a high‐speed rolling locomotion under constant humidity. Constructed from a single‐layer AG film, a biomacromolecule highly responsive to humidity,^[^ ^51^ ^]^ the Dualicalbot features a closed‐loop structure comprising two reversely spined helical strips connected by two semicircular ones. When placed on a humid surface, the special asymmetric structural design makes sure that the two helices alternately generate the required barycenter offset for the robot to roll, each actuating half a cycle, and thus an ultrafast rolling locomotion is obtained. We demonstrate through theoretical analysis and experiments that the optimal design, weighing only 29.5 mg, achieves a remarkable rolling speed of 5.8 BL s^−1^, 4.8 times faster than the fastest robot driven by a constant environment and surpassing the majority of untethered soft robots driven by modulated stimuli. Moreover, it can carry a payload up to 100% of its own weight, and detect acidic environments it rolls through by discoloring.

## 2 Results

### 2.1 Structural Design of the Dualicalbot

**Figure 1:**
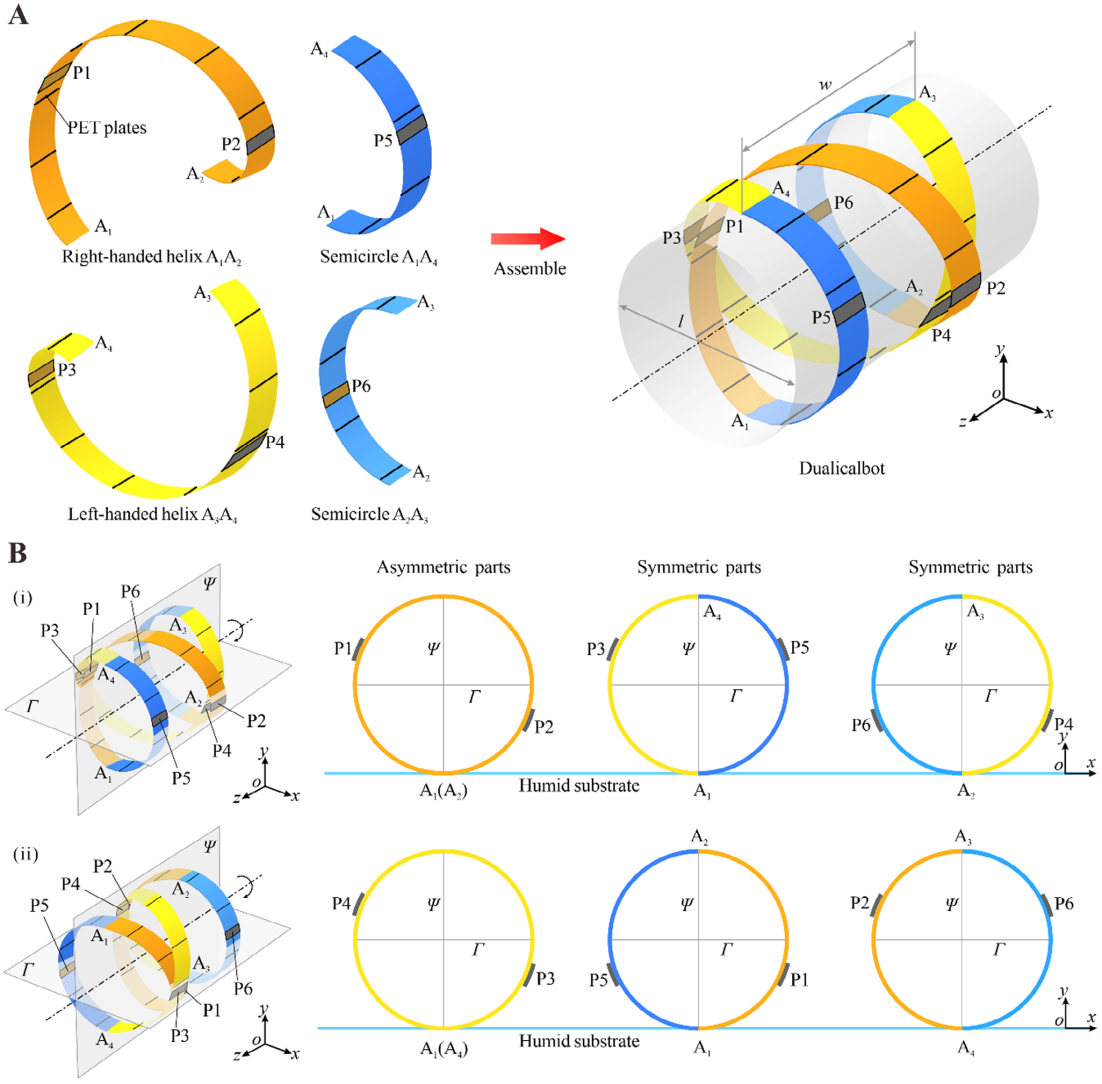
Structural design of the Dualicalbot. A) The Dualicalbot is assembled by two helical strips A_1_A_2_ and A_3_A_4_, and two semicircular strips A_1_A_4_ and A_2_A_3_. PET plates are placed on both sides of each strip, and six tapes P1 – P6 are attached to the strips at prespecified positions. B) The specific positions of the tapes with respect to the reference planes *Γ* and *Ψ*, which are parallel to the *xoz* and *yoz* coordinate planes, respectively, and intersecting the geometric center of the robot. i) is the first half cycle, and ii) is the second half cycle after rolling 180°.

The Dualicalbot, shown in **Figure** **1**A, consists of a right‐handed helix strip A_1_A_2_ and a left‐handed helix strip A_3_A_4_, which are connected by two semicircle ones A_1_A_4_ and A_2_A_3_ to form a closed‐loop structure on a cylindrical surface with a body length of *l* and a width of *w*. Slender polyethylene terephthalate (PET) plates, denoted as black lines, are attached to both sides of each strip at intervals to ensure a unidirectional bending deformation about the cylinder axis.^[^ ^34^ ^]^ Additionally, six tapes P1‐P6 are attached to the outer surface of the robot, two tapes on each helix and one on each semicircle, denoted as grey rectangles. In order to make this double‐helical structure to generate periodic rolling motion along the positive *x*‐axis, its symmetry should be broken to achieve a barycenter offset. Hence, the specific positions of these tapes are crucial for the periodic rolling of the robot as illustrated in Figure 1B. Projecting the robot onto the *xoy* plane, three circles can be obtained as shown in Figure 1B(i). For the right‐handed helix A_1_A_2_, tapes P1 and P2 are respectively on the upper left and lower right side of the reference plane *Γ* to form an asymmetric distribution, while P3 on the left‐handed helix A_3_A_4_ and P5 on the front semicircle A_1_A_4_ as well as P4 on helix A_3_A_4_ and P6 on the rear semicircle A_2_A_3_ both form symmetric distribution about plane *Ψ*. When placing the robot on a humid substrate, the film of the right‐handed helix A_1_A_2_ on the lower side of plane *Γ* that faces toward the substrate will absorb humidity and generate local bending deformation. Due to the existence of tape P2, the left side will deform more than the right side. Therefore, the attachment of tape causes an asymmetric local deformation in A_1_A_2_, resulting in a barycenter offset to drive the robot to roll toward right. On the contrary, the asymmetric deformation of the left‐handed helix A_3_A_4_ is counteracted by that of the semicircular strips A_1_A_4_ and A_2_A_3_, as the tapes P3 and P5 are symmetric about plane *Ψ* and so are P4 and P6. Thus A_3_A_4_ does not provide driving moment at this stage. After half of a rolling 180° (Figure 1B(ii)), the barycenter offset is generated by the asymmetric deformation of helix A_3_A_4_ due to the asymmetric distribution of tapes P3 and P4 while that of helix A_1_A_2_ is cancelled out again by semicircles A_1_A_4_ and A_2_A_3_. Therefore, the two helices alternatively drive the robot in each cycle, leading to a periodic unidirectional rolling motion as shown in Movie S1 (Supporting Information). Compared with the existing single‐helical robots driven by constant stimuli,^[^ ^19^ ^]^ which typically contain three stages in each cycle, that is, shape deformation to generate the required driving moment, rolling motion, and shape recovery for the next cycle, the double‐helical structure does not require the shape recovery stage as one helix has enough time to recover to its original shape while the other one drives the robot, thus leading to faster motion speed.

### 2.2 Theoretical Model

**Figure 2:**
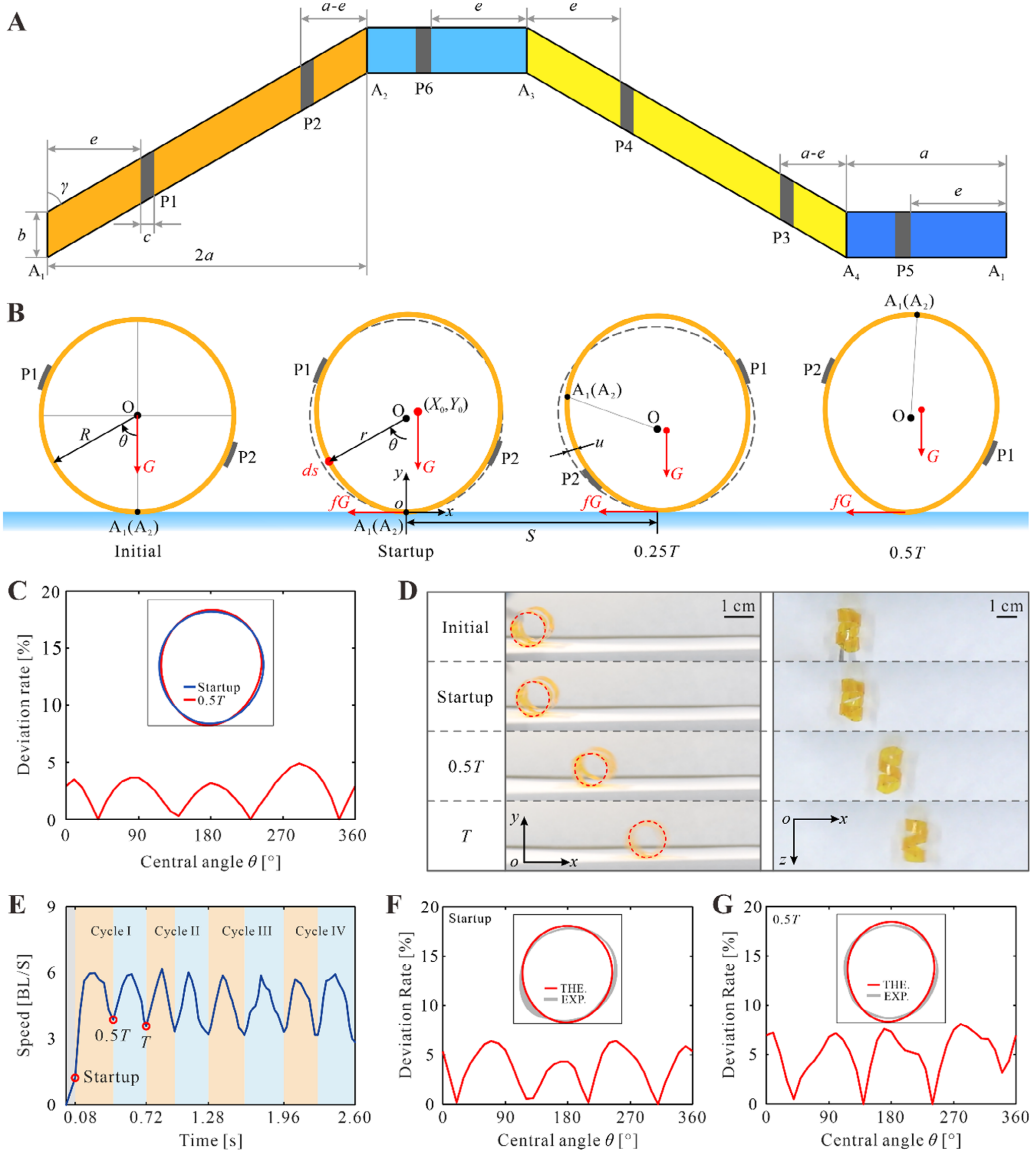
Theoretical modeling and experimental validation of the Dualicalbot. A) Geometric parameters of the Dualicalbot. The corrugated strip is a flattened double‐helical structure by cutting at the point A_4_. The colors of the four segments correspond to Figure 1A. B) Theoretical configurations of the right‐handed helix A_1_A_2_ projected on the *xoy* plane during the first half cycle. C) The configurations comparison and deviation of helix A_1_A_2_ at the end of the startup stage and at 0.5*T*, which are obtained from the theoretical model. D) The side view and top view snapshots of the Dualicalbot captured from Movie S2 (Supporting Information) in the startup stage and the first rolling cycle. E) The speed (BL s^‐^^1^) versus time (s) of the Dualicalbot in the first four locomotion cycles in Movie S2 (Supporting Information). F‐G) Comparison of the experimental and theoretical configurations at the end of the startup stage and at 0.5*T*, respectively, and the shaded bands are the experimental results of three tests.

To better explain the rolling behavior of the Dualicalbot, a theoretical model is established to obtain its configuration and energy variation during locomotion. As shown in **Figure** **2**A, the two helices, which are obtained from two parallelogram strips, are defined by the height 2*a*, width *b*, helical angle *γ*, and thickness *μ*. The two semicircles, which are obtained from two rectangular ones, have a length of *a*, a width of *b*, and the same thickness *μ*. The positions of tapes P1 – P6 with a width of *c*, can be determined by a single parameter *e*, which is the distance between the tapes and the edges A_1_ – A_4_. Based on the geometric parameters, the body length *l* and width *w* of the Dualicalbot can be calculated as *l* = 2*a*/π and *w* = *b* + 2*a*/tan γ. In addition, to avoid physical interference of neighboring strips, *a*, *b*, and *γ* should satisfy the relationship *b*(1 + sin γ)tan γ < 2*a*.

As illustrated above, the robot is driven by the right‐handed helix A_1_A_2_ in the first half cycle, and its deformation and rolling behaviors are shown in Figure 2B. Initially, the offset of barycenter occurs with the asymmetric humidity absorption as mentioned above in Figure 1B(i). When the moment caused by the gravity force is greater than that by the frictional force, the robot begins to roll toward right, and this stage is denoted as the startup stage. Based on the responsive mechanism and actuation capacity of the AG film (Note S1, Supporting Information), the final configuration of this stage can be obtained by the conservation equation of energy in a duration of *T*_0_

(1) $$\Delta E_{H}\left(T_{0}\right)=\int_{0}^{2\pi R}\Delta E_{B}\left(s\right)ds+\int_{0}^{2\pi R}\Delta E_{G}\left(s\right)ds$$

where *E*_H_, *E*_B_, and *E*_G_ are respectively the humidity energy, bending strain energy, and gravitational potential energy, and *R* and *s* are respectively the initial radius and arc length of helix A_1_A_2_ projected onto the *xoy* plane, see more details in Note S2 and Figure S1 (Supporting Information).

With the deformed configuration being obtained, the critical condition for the robot to roll is that the driving moment caused by the barycenter offset is equal to that caused by the frictional force, that is,

(2) $$GX_{0}\left(T_{0}\right)=fGY_{0}\left(T_{0}\right)$$

where *X*_0_ and *Y*_0_ are the barycenter position of helix A_1_A_2_ after deformation, *G* is the gravity of the Dualicalbot (Note S2, Supporting Information), and *f* is the friction coefficient determined as 0.5556 from the experiment (Note S3; Figure S2, Supporting Information). Then the time of the startup stage *T*_0_ can be obtained by Equations (1) and (2).

After the startup stage, the robot will roll following the direction of the barycenter offset. During motion, it continuously absorbs humidity energy and changes configuration accordingly. When it rolls by a distance of *S*, the configuration at *S* + *dS* is determined by the current configuration at *S*, the rigid‐body rolling motion during *dS*, as well as the deformation caused by humidity absorption during this process. Thus, the recurrence relation of the radius of helix A_1_A_2_ projected on the *xoy* plane, *r*, can be obtained as

(3) $$r\left(s,S+dS\right)=r\left(s,S\right)+\frac{\partial r\left(s,S\right)}{\partial s}-u\left(s,S,dS\right)$$

where ∂*r*(*s*, *S*)/∂*s*describes the rigid‐body rolling, and *u*(*s, S, dS*) is the radical displacement caused by humidity absorption during *dS*. Different from the startup stage, in addition to the humidity energy *E*_H_, bending strain energy *E*_B_, and gravitational potential energy *E*_G_, the kinetic energy of the robot *E*_K_ and the energy dissipated by friction *E_f_* also need to be considered. Then the time *dt(S)* for the rolling distance *dS* can be solved by simultaneously considering the energy conservation equation and the dynamic Equation (4)

(4) $$\left\{\begin{array}{c} dE_{H}=dE_{B}+dE_{G}+dE_{K}+dE_{f}\\ M_{G}-M_{f}=I\dot{\omega } \end{array}\right.$$

where ***M***_G_ and ***M*** *_f_* are the gravity moment and the friction moment, respectively, *I* is the rotational inertia, and $\dot{\omega }$ is the angular acceleration, details in Note S2 (Supporting Information). Combining Equations (3) and (4), the time required for a rolling distance of *S*, and the corresponding configuration can be solved.

Comparing the configurations of the robot after rolling half a cycle (*S* = π*R*) and that at the end of the startup stage (*S* = 0), we find that they are approximately the same as shown in Figure 2C with the largest deviation less than 5% of the corresponding radius at the position defined by central angle *θ* (details in Note S4, Supporting Information). The reason is that the increase in humidity energy is nearly completely dissipated by the friction during rolling. This finding thus proves that the actuating helix can restore to the startup configuration after the first half cycle. In the second half cycle, the other helix will undergo the same deformation and restoration, and therefore the robot is capable of a periodic rolling motion. The cycle time *T* can be calculated by doubling the time for half a cycle as $T=2\int_{0}^{\pi R}dt(S)$, and the motion speed measured by body length can be obtained as *v* = π / *T*.

To validate the theoretical model, a Dualicalbot with parameters *a* = 18 mm, *b* = 6 mm, *γ* = 60°, *μ* = 15 µm, *c* = 1.5 mm, and *e* = 9 mm was fabricated and tested at the relative humidity (*RH*) of 70%. It can be seen from the side view and top view snapshots in the first cycle in Figure 2D and Movie S2 (Supporting Information) that the robot rolled continuously on the humid substrate. The history of experimental locomotion speed with respect to time in Figure 2E indicates that a periodic rolling motion is successfully generated. And the speed curves of the first and second half of each cycle are also very close, proving that the two helixes alternatively drive the robot in a cycle. Furthermore, the configurations of the robot at the end of the startup stage and at 0.5*T* are also extracted from experiments and compared with theoretical predictions in Figure 2F,G. A good match with the largest error of less than 8% is obtained, indicating that the theoretical model is capable of capturing the motion characteristics of the robot.

### 2.3 Parametric Analysis

**Figure 3:**
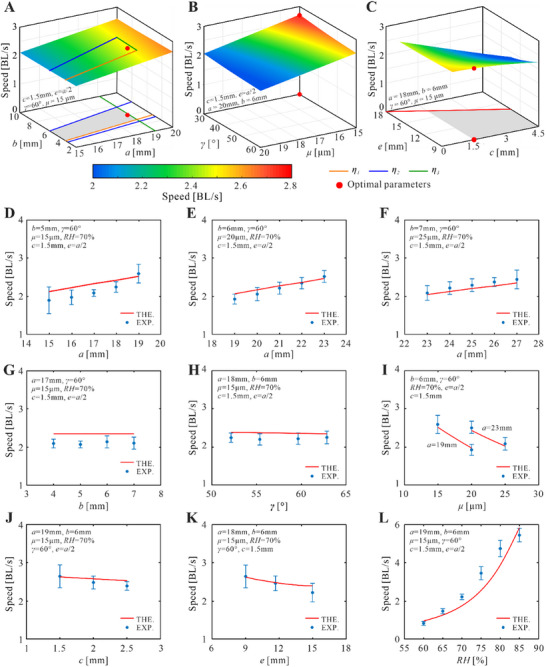
Parametric analysis of the Dualicalbot. A–C) The theoretical predictions of the speed vary with different values of geometric parameters. A) is the effect of the strip length *a* and width *b* on speed, B) is of the helical angle *γ* and the strip thickness *μ*, and C) is of the tape width *c* and the tape position *e*. D‐L) The experimental validation of the theoretical results. D–F) show the relationships between the rolling speed and *a*, and G‐L) are the relationships between the rolling speed and *b*, *γ*, *μ*, *c*, *e*, and *RH*, respectively. The red lines are the theoretical results, and the blue lines are obtained from experiments, which contain the error bars of at least four measurements.

Having demonstrated the motion characteristics of the robot, we subsequently conduct a parametric study based on the theoretical model to unveil the effects of geometric and environmental parameters. First consider the geometric parameters *a*, *b*, *γ*, and *μ* that determine the overall size and stiffness of the robot. The variation of the rolling speed with respect to *a* and *b*, in which *γ* = 60° and *μ* = 15 µm, is shown in **Figure** **3**A, and that with respect to *γ* and *μ*, in which *a* = 20 mm and *b* = 6 mm, in Figure 3B. All the other parameters are selected as *c* = 1.5 mm, *e* = *a*/2, and *RH* = 70%. The theoretical model predicts that the rolling speed increases approximately linearly with the length of the strip *a*, while the width of the strip *b* and the helical angle *γ* have little influence on the speed. This is because according to the expression of the body length *l* mentioned above, *a* determines the cross‐sectional size of the robot. With the increase in the cross‐sectional size, the bending curvature of the AG film is reduced, and thus it becomes easier for the robot to bend upon absorbing humidity. Since the bending strain energy dominates in the total energy of the robot, typically accounting for over 81.35% of the total energy (Note S5, Supporting Information), this leads to a reduced deformation time and thus a faster rolling speed. On the other hand, *b* and *γ* only influence the width of the robot without changing the curvature of the AG film, and thus have little effect on the rolling speed of the robot. Finally, the thickness *μ*, which determines the bending stiffness of the AG film, also has a strong correlation with the rolling speed. A thinner strip is easier to bend, and thus makes the robot deform more rapidly so as to achieve a faster speed.

Moreover, the geometric parameters of the tapes P1 – P6 influence locomotion capacity as well. Thus, we illustrate the variation of the rolling speed with respect to the tape width *c* and the tape location *e*, as shown in Figure 3C, in which *a* = 18 mm, *b* = 6 mm, *γ* = 60°, *μ* = 15 µm, and *RH* = 70%. The theoretical result indicates that the rolling speed increases with the reduction in either *c* or *e*. This is owing to that *c* determines the area of the AG film shielded by the tapes. A smaller value of *c* leaves more effective material for humidity absorption, thus leading to a higher rolling speed. Meanwhile, *e* describes the distance between the tape and the contact point of the robot. Taking tape P2 as shown in Figure 1B(i) as an instance. The smaller the value of *e*, the further away P2 is from the contact point A_2_. Since P2 can generate the asymmetric deformation for driving only when it is on the right‐hand side of the contact point, a minimal *e* means that it can provide driving moment for up to a quarter of a cycle, thus leading to a faster speed.

To further validate the theoretical relationship, we fabricated a series of robots with different combinations of geometric parameters and tested them at different relative humidity. Generally, the experimental results in Figure 3D–K match reasonably well with the theoretical predictions.

Theoretically, the geometric parameters can be arbitrary selected as long as the geometric constraint in Equation (2) is satisfied. Through various experiments on the Dualicalbot with different parameters, nevertheless, we find that these parameters should be limited in a certain range to ensure that the robots deform and roll steadily in a periodic manner. Specifically, the following three limiting parameters are defined as follows.

The first one is the ratio of the length to the width of the robot η_1_ = *l*/*w*. The upper bound of this ratio is experimentally determined as 0.45 at three different film thicknesses (15 µm, 20 µm, and 25 µm). If η_1_ exceeds 0.45, the robot is too slim and prone to fall aside as shown in Figure S3A (Supporting Information).

The second one is the ratio of the width to the screw pitch of the two helices η_2_ = *b*sin γtan γ/(2*a*). This parameter should be in the range from 0.17 to 0.31. If η_2_ is lower than 0.17, the robot cannot stably maintain its helical shape and becomes despiralized after a few cycles (shown in Figure S3B, Supporting Information). Meanwhile, when η_2_ is larger than 0.31, a substantial area of the driving helix will be occluded by the other one from absorbing humidity. This cancels out the effect of the asymmetrically placed tapes, and leads to only deformation of the robot but no offset of gravitational barycenter (shown in Figure S3C, Supporting Information).

The third limiting parameter is η_3_ = 2*a*/(πμ), which defines the relationship between the body length of the robot and the thickness of the AG film, and its range should be between 0.58 × 10^3^ and 0.81 × 10^3^. This parameter dictates how easily the film can be bent. If η_3_ is too large, the robot is overly deformed by humidity absorption and despiralized before it starts to roll (shown in Figure S3D, Supporting Information). Meanwhile, the robot is difficult to generate the required deformation if η_3_ is too small (shown in Figure S3E, Supporting Information).

Additionally, we find out that the asymmetrical deformation is not large enough to generate the required barycenter offset if the tape width *c* is smaller than 1.5 mm in experiments. Therefore, the minimum value of *c* is 1.5 mm.

Apart from the geometric parameters, the relative humidity *RH* also has significant effects on the motion speed as it determines the humidity absorption rate *ξ* according to Equation S1 (Supporting Information). Thus, we also conducted experiments on the Dualicalbot with *a* = 19 mm, *b* = 6 mm, *γ* = 60°, *μ* = 15 µm, *c* = 1.5 mm, and *e =* 9.5 mm at varying *RH* from 55% to 90% at an interval of 5%. It is found that the robot can roll steadily between *RH* = 60% and *RH* = 85%, and the experimental results match the theoretical predictions reasonably well as presented in Figure 3L. When *RH* reaches 90%, the robot is excessively deformed and becomes despiralized (shown in Figure S3F, Supporting Information). Meanwhile, if the *RH* is reduced to 55%, the robot cannot accumulate enough deformation to initiate the rolling motion (shown in Figure S3G, Supporting Information).

### 2.4 Performance Demonstrations

**Figure 4:**
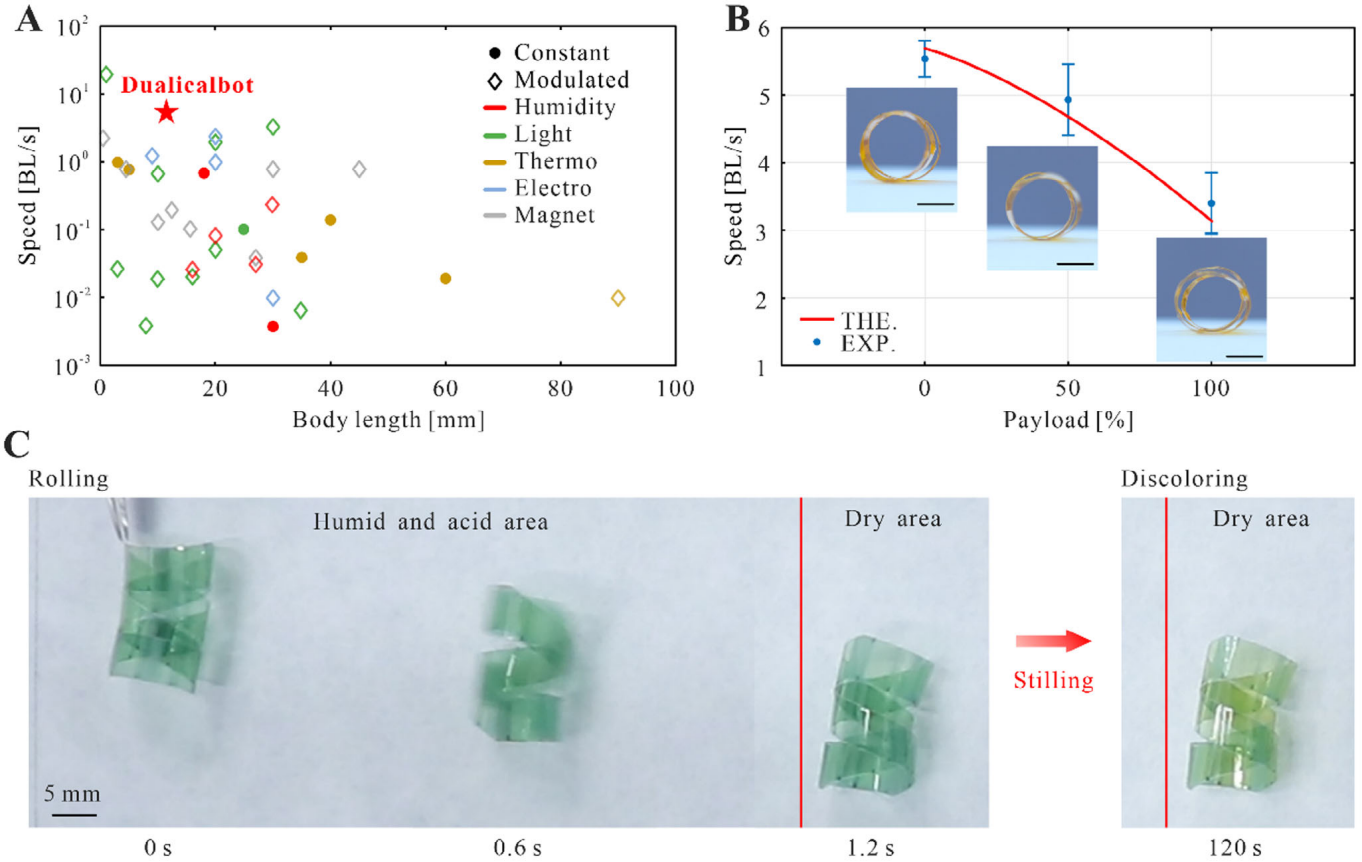
Performance demonstrations of the Dualicalbot. A) Ashby plot of the maximum locomotion speed (BL s^−1^) versus the body length (mm) in the reported untethered soft robots made from stimuli‐responsive materials. B) The relationship between rolling speed and payload of the Dualicalbot with optimal geometric and environmental parameters (scale bar: 6 mm). C) Experimental snapshots of the acid environment detection robot rolling over the humid and acid area, and then discoloring in the dry and neutral area.

Based on the above analysis, we can determine the optimal geometric and environmental parameters of the Dualicalbot as follows: *a* = 19 mm, *b* = 5 mm, *γ* = 60°, *μ* = 15 µm, *c* = 1.5 mm, *e =* 9.5 mm, and *RH* = 85%. A robot with those parameters, which weighed 29.5 mg (Note S6, Supporting Information), was fabricated and tested, and a rolling speed of 5.8 BL s^−1^ (Movie S3, Supporting Information) was achieved. **Figure** **4**A shows a comparison of the relative speed (BL s^−1^) versus body length (mm) of untethered soft robots made from various stimuli‐responsive materials which are powered either by constant environments (marked as circles) or by modulated stimuli (marked as diamonds), details in Table S4 (Supporting Information). It can be seen that the Dualicalbot that is marked as a red star is faster by 4.8 times than the fastest reported robot driven by a constant environment (1 BL s^−1^), and outperforms the majority of soft robots driven by modulated environments. Furthermore, the life‐span of the Dualicalbot is primarily determined by that of the AG film which would saturate after many cycles of water absorption/desorption. Based on the fatigue test result of the film, details in Note S1 (Supporting Information), it can bear over 1000 cycles when the relative humidity is in the range of 60% to 90%.

Moreover, the loading capacity of the Dualicalbot has been analyzed as well by attaching additional PET plates on the opposite side of tapes P1 – P6 which weigh 15 mg (50.84% of self‐weight), 30 mg (101.69% of self‐weight), and 45 mg (152.54% of self‐weight), respectively, to the optimal robot as payload (details in Note S7; Figure S4, Supporting Information). The experimental result indicates that the rolling speeds of the robots with 15 mg and 30 mg payload are 4.9 BL s^−1^ and 3.4 BL s^−1^ respectively (Movie S4, Supporting Information), which are consistent with theoretical predictions as shown in Figure 4B. When the payload increases to 45 mg, the Dualicalbot is overly deformed by the payload and cannot roll forward. Therefore, it can be concluded that the robot can carry a payload of its own weight and still achieve a very fast rolling speed.

Additionally, the ability of the Dualicalbot to roll on an inclined surface is demonstrated. As validated by both theoretical modeling and experimental results (Note S8; Figure S13, Supporting Information), the robot can successfully roll over a slope with an inclination angle of 2°.

### 2.5 Acid Environment Detection

Detection of environments with special physical or chemical properties is a significant application scenario of soft robots. Taking advantage of the locomotion of the Dualicalbot and the stable chemical property of AG, which does not react chemically with common pH indicators, we develop a robot capable of detecting an acid environment. This is achieved by incorporating bromocresol green (BG), a sensitive acid‐base indicator, into the AG film so that the robot will discolor when it passes through an acid substrate (details in Experimental Section and Methods). Moreover, the optimal geometric parameters as shown in the previous section are adopted.

The experiment snapshots of the detecting robot are shown in Figure 4C and Movie S5 (Supporting Information). The robot, whose initial color is light green due to BG, rolls over the humid and acidic area and absorbs hydrogen ions during motion. The rolling speed is still 5.8 BL s^−1^, inferring that the inclusion of BG indicator does not affect its motion capability. When entering the dry area, the robot stays still and gradually discolors from light green to yellow during a period of 120 seconds, indicating that it has gone through an acid environment. As a control, the robot passing through a neutral humid area does not change color as shown in Movie S5 (Supporting Information). This result thus proves the function of acid environment detection of the Dualicalbot.

## 3 Conclusion

In this research, we integrate a double‐helical structure with the humidity responsive agarose film to develop an untethered soft robot named Dualicalbot that is capable of an ultrafast rolling locomotion under a constant humid environment. When the Dualicalbot absorbs humidity and deforms, an offset of the barycenter is generated to provide a driving moment for the robot to roll. A theoretical model is established to correlate the bending deformation of the film to the motion of the robot. It has been found out that the right‐handed and left‐handed helices alternatively drive the motion of the robot, and the robot returns to the same configurations every half a cycle, thus unveiling the underlying mechanism for the periodic locomotion. Moreover, the effects of the geometric parameters and environmental humidity have also been obtained and validated by experiments. Based on this, an optimal Dualicalbot, weighing only 29.5 mg, achieves a maximum rolling speed of 5.8 BL s^−1^, 4.8 times faster than the fastest soft robot driven by a constant environment, and surpassing most untethered soft robots driven by modulated ones. Moreover, it can roll with a payload of 101.69% of its self‐weight in a speed of 3.4 BL s^−1^. Finally, by dyeing its body with the acid indictor bromocresol green, we showcase that the robot can detect the acid environment it rolls past through discoloring.

Evidently, the same geometric design can be applied to the other stimuli‐responsive materials to generate the similar motion. More importantly, we expect that the framework of structural design and theoretical modelling presented in this paper will provide useful guidance for the design of soft robots based on stimuli‐responsive materials. Future research will focus on developing new soft robots with superior motion capability and expanding their functions serving various engineering applications.

## 4 Experimental Section

### Preparation of AG Films

The process to fabricate an AG film is summarized in Figure S5 (Supporting Information). First, 2 g of agarose powder (AG, Shanghai Macklin Biochemical Technology Co., Ltd) and 0.02 g of phenol red powder (PR, Beijing Solarbio Technology Co., Ltd., analytical reagent) as a dye for observation are dissolved in 60 ml of N,N‐dimethylformamide (DMF, Tianjin Concord Technology Co., Ltd., analytical reagent). And then the mixed solution is heated to 95 °C with a vigorous stirring of 800 rads per minute (rpm) by the magnetic heating agitator (HS‐5C; Zhejiang OAIC Science and Technology Instrument Co., Ltd.) until the AG powder is completely dissolved. After heating and stirring for ≈ 4 h, the DMF/AG solution is spread evenly on the chromatographic plate (HSGF254; Yantai Jiangyou Silicone Development Co., Ltd.) with the size of 180 mm × 180 mm, and is dried in a ventilated environment (35±10% RH, 25 ± 5 °C). After three days’ natural evaporation, the AG film is taken off and stored in dry‐sealing bags. The relationship between the thickness of a piece of AG film and the solution spread on a plate is shown in Table S1 (Supporting Information).

Moreover, in the demonstration of the acid environment detection, 0.01 g of bromocresol green (BG), a sensitive acid‐base indicator, is incorporated in the AG‐DMF mixed solution as the indicator instead of 0.04 g of phenol red during the manufacturing of the new AG films.

### Fabrication Process of the Dualicalbot

A simple method was developed for fabricating the Dualicalbot, which can be divided into the following steps (details in Movie S1, Supporting Information). First, PET plates and tapes are attached to the two rectangular AG strips and the two parallelogram ones. Second, these four strips are attached in designed order to form a corrugated strip. Third, the corrugated strip is connected end‐to‐end to become a closed loop. Fourth, one rectangle strip is constrained while the other is rotated for 360°, and each parallelogram strip will form a helical structure with one cycle naturally. Finally, the rectangle strip is stretched and then the closed loop will form the double‐helical structure under the action of internal stress.

### Experimental Setup

The experimental setup is shown in Figure S6 (Supporting Information). In order to provide a constant environmental humidity for the locomotion experiments of the Dualicalbot, an airtight control box with latex gloves is customized. The size of the box is 1600 mm × 1000 mm × 1200 mm (length × width × height), and the desiccant (allochroic silicagel) is used to maintain the humidity of the box to a constant 20% *RH*. To achieve locomotion, the robots with different geometric parameters are placed on wet filter paper (300 mm × 150 mm) above warm water in the vessel. The humidity of the filter paper can be adjusted by changing the temperature (2 to 61 °C) of the water. A RH sensor (± 2% RH, Anymetre TH21E, China) is placed on the substrate to measure the humidity. Moreover, the locomotion videos and photos of the Dualicalbot are recorded by a digital camera (LEICA SUMMARIT‐H, Germany). The relationship of rolling speed and time is obtained by the CAE software Tracker in a frame time of 0.02 s.

In the demonstration of the acid environment detection, the 50% formic acid solution is obtained by diluting 300 ml of 96% high‐purity formic acid solution (Shanghai Meiruier Biochemical Technology Co., Ltd) with 300 ml of pure water made by ultrapure water machine (HYP‐QX‐UP; Beijing Huiyipu Environmental Protection Technology Co., Ltd), as shown in Figure S7 (Supporting Information). In addition, the platform size of the left area is 200 mm × 100 mm and that of the right area is 100 mm × 100 mm.

## Conflict of Interest

The authors declare no conflict of interest.

## Author Contributions

C.X. and J.M. contributed equally to this work. Y.C. initiated the research. Y.C., C.X., J.M., and L.F. designed the structure of the robot. X.L. and L.Z. designed the film. C.X. and L.F. performed the experiments and analyzed the data. C.X. and J.M. set up the theoretical model. C.X., and J.M. drafted the manuscript. Y.C. and J.M. revised and finalized the manuscript.

## Supporting information



Supporting Information

Supplemental Movie 1

Supplemental Movie 2

Supplemental Movie 3

Supplemental Movie 4

Supplemental Movie 5

## Data Availability

All data needed to evaluate the conclusions in the paper are present in the paper and/or the Supporting Information. Additional data related to this paper may be requested from the authors.
