# Supplementary material for: An Ultra‐Fast Rolling Double‐Helical Robot Driven by Constant Humidity
Source: Adv Sci (Weinh). 2025 Apr 2;12(23):2500577. doi: 10.1002/advs.202500577 (PMC12199317; doi:10.1002/advs.202500577)
Supplement: Supplementary file 1 — Supporting Information [file ADVS-12-2500577-s006.docx]

Supporting Information

An Ultra-Fast Rolling Double-Helical Robot Driven by Constant Humidity

Chuhan Xu, Jiayao Ma, Lei Fu, Xinmeng Liu, Lei Zhang*, and Yan Chen*

C. Xu, J. Ma, L. Fu, Y. Chen

Key Laboratory of Mechanism Theory and Equipment Design of Ministry of Education, Tianjin University, 135 Yaguan Road, Tianjin, 300350, China.

School of Mechanical Engineering, Tianjin University, 135 Yaguan Road, Tianjin, 300350, China.

X. Liu, L. Zhang

Department of Biochemical Engineering, Frontier Science Center for Synthetic Biology and Key Laboratory of Systems Bioengineering (MOE), School of Chemical Engineering and Technology, Tianjin University, Tianjin, 300350, China.

L. Zhang

Haihe Laboratory of Sustainable Chemical Transformations, Tianjin, 300192, China.

C. Xu and J. Ma contributed equally to this work.

E-mail: [lei_zhang@tju.edu.cn](mailto:lei_zhang@tju.edu.cn) (L. Zhang), [yan_chen@tju.edu.cn](mailto:yan_chen@tju.edu.cn) (Y. Chen).

# This PDF file includes:

1. Note S1. Responsive mechanism and actuation capacity of the AG film.
2. Note S2. Theoretical model of the Dualicalbot.
3. Note S3. Measurement of the friction coefficient between the AG film and the filter.
4. Note S4. The definition of the deviation of different configurations.
5. Note S5. The variation of every kind of energy of the Dualicalbot during rolling.
6. Note S6. The mass composition of the Dualicalbot.
7. Note S7. The payload placement strategy of the Dualicalbot.
8. Note S8. Theoretical model and experimental validation of the Dualicalbot rolling on a slope.
9. Figures S1 to S13
10. Tables S1 to S5
11. Legends for Movies S1 to S5
12. Reference

# Note S1. Responsive mechanism and actuation capacity of the AG film.

- The Chemical Structure and Humidity Exchange Mechanism

As a biopolymer, AG possesses a substantial number of free hydroxyl groups on its surface.^[S1]^ When exposed to a humid environment, these hydroxyl groups exhibit strong humidity sensitivity, forming hydrogen bonds with oxygen atoms in water molecules. This process releases the chemical energy of the hydrogen bonds, which subsequently triggers hygroscopic expansion in the AG film, causing it to bend.^[S2]^ Upon moisture evaporation, the film undergoes desiccation shrinkage, leading to its recovery to the original shape. The chemical structure and humidity exchange mechanism are illustrated in Figure S8.

- The Water Absorption and Desorption Rates

In our previous research,^[S3]^ we investigated the water absorption and desorption rates of AG films with varying thicknesses and projected lengths under different relative humidity. The corresponding data are presented in Table S1, Supporting Information. Specifically, by measuring the curvature changes of the AG film with a length of *L*, a thickness of *μ*, and a width of 6 mm under different relative humidity *RH* ranging from 45% to 70% (in increments of 5%), the variations of their bending strain energy and gravitational potential energy can be obtained. Based on these energy changes and the duration of deformation, the water absorption rate correlated with 𝐿, 𝜇, and *RH*, was obtained. Similarly, the water desorption rate was measured by placing the deformed film on a dry surface and recording the time required for it to revert to its original shape.

- The Fatigue Life-span

We evaluated the fatigue life-span of the films with different thicknesses under varying humidity conditions. As depicted in Figure S9A, the experimental setup consisted of a stepper motor rotating at a speed of 8 seconds per cycle, which was connected to a plastic disc with a 32 mm diameter hole covered by a filter. A vessel containing water at different temperatures was used to modulate the relative humidity from 60% to 90% (in increments of 10%). The AG films, with thicknesses of 15 μm, 20 μm, and 25 μm, were secured horizontally on an arm. The deformation of the film with a thickness of 15 μm under a relative humidity of 90% was captured by the camera as shown in Figure S9B. The experimental results, presented in Figure S10, indicate that the AG film can maintain stable actuation performance for over 1000 cycles, with nearly no influence from variations in relative humidity and film thickness.

- The Payload Capacity

The payload capacity of the AG film has been assessed as well. As shown in Figure S11, several PET strips (1 mm × 6 mm × 50 μm) were attached to the free end of the AG film (6 mm × 18 mm × 15 μm) as payloads. The film was then exposed to a piece of filter with a relative humidity of 90%. By analysing the actuation strain under different payloads relative to the self-weight of the film, the payload capacity of the AG film was determined, as shown in Figure S12.

# Note S2. Theoretical model of the Dualicalbot.

To simplify the theoretical model, the following assumptions are established. First, the material facing the humidity surface absorbs humidity energy at a constant rate *ξ* related to the relative humidity *RH*, film thickness *μ*, and the projected length of the water absorption area *L*, and it follows

$$\xi\left(RH,\mu,L\right)=k_{1}e^{k_{2}RH}\mu^{0.5}L^{1.5}$$

where *k*_1_ = 4.424×10^-5^ kg·s^-3^ and *k*_2_ = 9.994 are two material constants.^[S3]^ Second, the water desorption of the material is ignored since it is much slower than the absorption rate as mentioned in Note S1. In other words, the robot does not lose humidity energy during motion. Third, only the bending strain energy of the AG film is considered since it is much larger than that caused by stretching, shearing, and twisting. And fourth, the tapes and PET plates are assumed to be uniformly distributed on the AG film in the calculation of the gravity of the robot, and have no effect on the bending stiffness of the film.

In order to solve the duration of the startup stage and the configuration of the Dualicalbot at the end of this stage, the right-handed helix A_1_A_2_ is virtually cut into three segments as shown in Figure S1, A_1_J, J'K' and KA_2_ where edges J, K, J', and K' are free. A_1_J and KA_2_ first deform without constraint due to water absorption, and then join J'K' to obtain the final configuration by applying boundary conditions at edges J (J') and K (K').

First consider the free deformation of A_1_J and KA_2_. Before deformation, the radius of A_1_J can be expressed as

$$R=\frac{l}{2}=\frac{a}{\text{π}}$$

and the barycentric coordinates at any point of A_1_J are

$$\left\{\begin{matrix} x\left(s\right)=R\sin\frac{s}{R} \\ y\left(s\right)=R\cos\frac{s}{R} \end{matrix}\right.\text{ }s\in[0,\frac{\text{π}l}{4}]$$

where *s* is the arc length starting at point A_1_, i.e., the contact point of the robot with the humidity surface.

When on the humidity surface, A_1_J will deform by absorbing humidity energy which is fully transformed into bending strain energy and gravitational potential energy. If we denote the radius of any infinitesimal arc *ds* in A_1_J as *r*^*^(*s*), in which ^*^ indicates that A_1_J is deformed but not yet connected to J'K', the central angle of the infinitesimal arc, *θ*^*^(*s*), and the central angle of the arc from 0 to *s*, *φ*^*^(*s*), can be respectively expressed as

Then the barycentric coordinates of any point in A_1_J are

Using the same procedure, the deformed configuration of KA_2_ can also be defined. Notice that the area shielded by tape P2 is undeformed, so the radius at any point is

$$r^{*}\left(s\right)=\left\{\begin{matrix} \begin{matrix} r^{*}\left(\text{π}l-s+ds\right)\text{ }s\in(\frac{3\text{π}l}{4},\text{π}l-e] \\ R\text{ }s\in(\text{π}l-e,\text{π}l+c-e] \end{matrix} \\ r^{*}\left(\text{π}l-s+ds\right)\text{ }s\in(\text{π}l+c-e,\text{π}l] \end{matrix}\right.$$

And the barycentric coordinates at any point of KA_2_ before and after deformation can be respectively solved by Equation (S5) and Equation (S6).

Based on the barycentric coordinates and radius, the changes in the humidity energy $\Delta E_{\text{H}}^{*}$, the bending strain energy $\Delta E_{\text{B}}^{*}$, and the gravitational potential energy $\Delta E_{\text{G}}^{*}$, of any infinitesimal arc *ds* in A_1_J and KA_2_ can be respectively obtained as

$$\left\{\begin{matrix} \Delta E_{\text{H}}^{*}\left(s\right)=T_{0}\xi(RH,\mu,\int_{0}^{\text{π}l/4} \cos\theta^{*}\left(s\right)ds+\int_{\text{3π}l/4}^{\text{π}l} \cos\theta^{*}\left(s\right)ds) \\ \Delta E_{\text{B}}^{*}\left(s\right)=\frac{Eb\mu^{3}}{48}[\frac{1}{r^{*}\left(s\right)^{2}}-\frac{1}{R^{2}}]ds \\ \Delta E_{\text{G}}^{*}\left(s\right)=\frac{G}{3\text{π}l}[y\left(s\right)-y^{*}\left(s\right)]ds \end{matrix}\right.$$

where *T*_0_ is the duration of the startup stage, *E* is the Young’s modulus of the AG film, and *G* is the gravity of the robot as follows

$$G=\left(6\rho_{\text{f}}ab\mu+10\rho_{\text{t}}cb\mu_{\text{t}}+n_{\text{p}}\rho_{\text{p}}w_{\text{p}}b\mu_{\text{p}}\right)g$$

in which $\rho_{\text{f}}$, $\rho_{\text{t}}$, and $\rho_{\text{p}}$ are respectively the density of the AG film, tape, and PET plate, *μ*, $\mu_{\text{t}}$, and $\mu_{\text{p}}$ are respectively the thickness of the AG film, tape, and PET plate. $n_{\text{p}}$ is the number of the PET plate, and $w_{\text{p}}$ is the width of the PET plate.

According to the conservation of energy, the following equation applies

$$\Delta E_{\text{H}}^{*}\left(s\right)=\Delta E_{\text{B}}^{*}\left(s\right)+\Delta E_{\text{G}}^{*}\left(s\right)$$

from which the deformed configurations of A_1_J and KA_2_ without constraints at boundaries J and K can be derived.

Next, we will join A_1_J and KA_2_ with J'K' to form the final shape by applying the boundary conditions that the radius and curvature at the edges J' and J should be respectively the same, and so are those at K' and K. Considering the symmetric boundary condition of J'K', there will be a shear force *F*_J_ and a moment *M*_J_ at edge J', while the force and moment at edge K' are *F*_K_ and *M*_K_, and the corresponding counteracting forces and moments will be applied to J and K as shown in Figure S1. Applying the equilibrium of force and moment for J'K', it is easy to work out that *F*_J_ = *F*_K_ and *M*_J_ = *M*_K_. Then the radial displacement and radius of curvature of the infinitesimal arc can be derived by the virtual work principle as

$$\left\{\begin{matrix} u_{\text{A}_{2}\text{J}}^{*}(s)=\frac{M_{\text{J}}^{2}}{6\text{π}REI_{z}}(2\text{π}Rs-2s^{2}+\text{π}^{2}R^{2}) \\ r_{\text{A}_{2}\text{J}}(s)=r_{\text{A}_{2}\text{J}}^{*}(s)+u_{\text{A}_{2}\text{J}}^{*}(s) \end{matrix}\right.\text{ }s\in(0,\frac{\text{π}l}{4})$$

where *I*_z_ is the inertia moment of the AG film.

Using the same procedure, the radial displacement and radius of curvature of any infinitesimal arc in J'K' and KA_2_ can also be calculated as

$$\left\{\begin{matrix} u_{\text{J'K'}}^{*}(s)=\frac{M_{\text{J}}^{2}[6(s-2c)(s-\text{π}R)-(2c-2s+\text{π}R)^{2}]}{6(2c/R-\text{π})REI_{z}} \\ r_{\text{J'K'}}(s)=R-u_{\text{J'K'}}^{*}(s) \end{matrix}\right.\text{ }s\in[\frac{\text{π}l}{4},\frac{\text{3π}l}{4}]$$

$$\left\{\begin{matrix} u_{\text{KA}_{3}}^{*}(s)=\frac{-M_{\text{J}}^{2}[6(2c-2s+3\text{π}R)^{2}\text{+}(s-2\text{π}R)(2c-s+\text{π}R)]}{36(2c/R-\text{π})REI_{z}} \\ r_{\text{KA}_{3}}(s)=r_{\text{KA}_{3}}^{*}(s)+u_{\text{KA}_{3}}^{*}(s) \end{matrix}\right.\text{ }s\in(\frac{\text{3π}l}{4},\text{π}l]$$

Subsequently, we apply the boundary conditions at J and J', and K and K' that the two pairs of edges can be respectively connected and the curvature is continuous, which leads to

$$\left\{\begin{matrix} r_{\text{A}_{1}\text{J}}(\frac{\text{π}l}{4})=r_{\text{J'K'}}(\frac{\text{π}l}{4}) \\ r_{\text{KA}_{2}}(\frac{\text{3π}l}{4})=r_{\text{J'K'}}(\frac{\text{3π}l}{4}) \end{matrix}\right.$$

Then the force *F*_J_ and moment *M*_J_ can be solved from Equation (S13), which can then be substituted into Equation (S10), Equation (S11), and Equation (S12) to get the final deformed configuration of the entire helix which can be defined by its radius.

With the deformed configuration being obtained, the offset displacement of the barycenter can be expressed as

$$\left\{\begin{matrix} X_{0}=\int_{0}^{\text{π}l} x\left(s\right)ds \\ Y_{0}=\int_{0}^{\text{π}l} y\left(s\right)ds \end{matrix}\right.$$

And the final configuration and the duration of the startup stage can be obtained by the critical condition introduced in the main text.

During the rolling stage, the radius *r* and radical displacement *u* of the recurrence Equation (3) in the main text can be obtained by the same method as the startup stage. Moreover, in the energy conservation equation, the kinetic energy can be expressed as

$$E_{\text{K}}=\frac{G}{2g}(\frac{dS}{dt})^{2}+\frac{I}{2r^{2}}(\frac{dS}{dt})^{2}$$

Based on the Newton-Euler Equation

The resultant moment contains the gravity moment *M*_G_ and the friction moment *M_f_*, the Coriolis term ***ω*** × (*I****ω***) = 0 due to the circular cross-section of the Dualicalbot, and the angular acceleration

The dynamic equation can be expressed as Equation (4) in the main text.

# Note S3. Measurement of the friction coefficient between the AG film and the filter.

To obtain the friction coefficient between the AG film and the filter, a dry filter is attached to a PMMA plate, and a piece of AG film is placed at the edge of the filter. Then, this edge is lifted by putting several glass slides under the endpoint of PMMA and the plate becomes a tilt slope. The quantity of glass slides is increased until the double-helical robot rolls opportunely. According to the numbers *n* and the height *h* of the glass slides and the length *l* of the plate, the friction coefficient *f* can be expressed as

$$f=\sin\frac{nh}{l}$$

In this experiment, we kept *h* = 1 mm and added the number of the glass slides. Based on the results of three time of experiments, the AG film began to slip when *n* = 6 and the length *l* was measured as 12.354 mm. Thus, the friction coefficient *f* could be obtained by Equation (S17) as 0.5556.

# Note S4. The definition of the deviation of different configurations.

To evaluate the difference of the configurations obtained from theoretical model or experiment snapshots, we divide the two circular cross sections in *xoy* plane, which need to be compared, evenly into 72 segments, then the deviation rate of every segment is defined as

$$\Delta=\frac{\left|r_{1}-r_{0}\right|}{r_{0}}$$

where *r*_1_ and *r*_0_ are the segment radius of each section, respectively. And the calculation results of the deviation are shown in Figure 2C, 2F, and 2G. Moreover, to obtain the configurations from experimental snapshots, we capture the outline of the front cycle according to the different colors of the Dualicalbot (yellow) and the background filter (white), and the center line of the yellow part is defined as the average result.

# Note S5. The variation of each energy of the Dualicalbot during rolling.

In the main text, we illustrate that the humidity energy *E*_H_ should transform into the bending strain energy *E*_B_, the gravitational potential energy *E*_G_, the kinetic energy *E*_k_, and the energy dissipated by friction *E_f_* during rolling. According to the calculated result of the theoretical model, the variation of *E*_B_, *E*_G_, *E*_k_, and *E_f_* are respectively 2.03 × 10^-7^ J, 2.95 × 10^-8^ J, 2.24 × 10^-9^ J, and 1.18 × 10^-8^ J when the robot rolls from 0 BL to 0.01 BL, which the bending strain energy accounts for over 81.35% of the total energy. Moreover, the magnitude of every energy does not change significantly throughout the locomotion, thus we can obtain that the bending strain energy dominates the total energy of the robot.

# Note S6. The mass composition of the optimal Dualicalbot.

The total weight of the optimal Dualicalbot is 29.5 mg, which contains 4 AG strips weighting 15.2 mg, 4 tapes (5 mm × 1.5 mm × 0.02 mm) for asymmetric bending and 4 tapes (5 mm × 3 mm × 0.02 mm) for connection weighting 6.4 mg, and 22 PET plates (5mm × 0.5 mm × 0.05 mm) weighting 7.9 mg.

# Note S7. The payload placement strategy of the Dualicalbot.

To measure the payload capacity of the Dualicalbot, we attach PET plates on the back of every tape for asymmetric bending as extra loading which is shown in Figure S3, the size of the PET plate is equal to that of the tape (5 mm × 1.5 mm) and its thickness is 0.075 mm. Specifically, we attach double layers of PET plates, which weigh 15 mg approximately, on the back of every tape for asymmetric bending as 50.84% self-weight payload of Dualicalbot. Similarly, four layers and six layers of PET plates are attached as 101.69% and 152.54% self-weight payload respectively.

# Note S8. Theoretical model and experimental validation of the Dualicalbot rolling on a slope.

As shown in Figure S13A, different from rolling on a flat surface, when the Dualicalbot rolls on a slope, the projection of gravity onto the slope creates an additional resistance moment, and the gravity moment *M*_Gs_, which is also the driving moment, can be modified as

$$M_{\text{G}s}=M_{\text{G}}(1-\sin\gamma)$$

where *M*_G_ is the gravity moment on a flat surface, and *γ* is the tilted angle of the slope. Simultaneously, the frictional resistance torque *M_f_*_s_ is determined as

$$M_{fs}=M_{f}\cos\gamma$$

where *M_f_* is the frictional resistance torque during rolling on a flat surface. The condition for the robot to successfully roll up the slope is given by

$$M_{\text{G}s}=M_{fs}$$

from which the maximum allowable tilted angle can be derived as 2.32°.

Furthermore, we conducted rolling experiments on a slope. As shown in Figure S13B, The Dualicalbot achieves to roll onto a slope with the inclination angle of 2° under optimal parameters.

# Figures S1 to S13


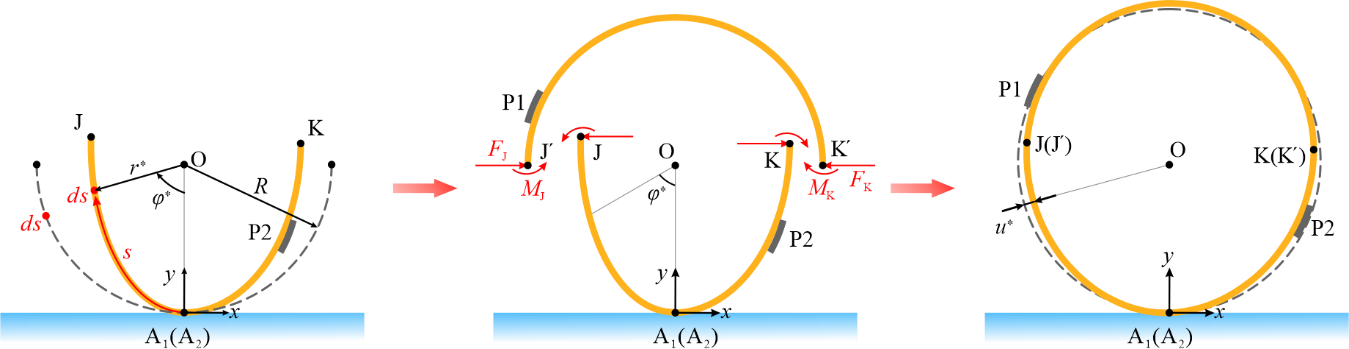


Figure S1. The analytical steps of the deformation of helix A_1_A_2_ at the startup stage.


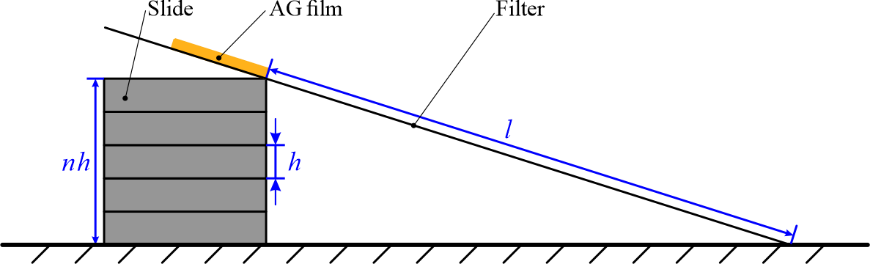


Figure S2. Setup of the experimental test of the friction coefficient.


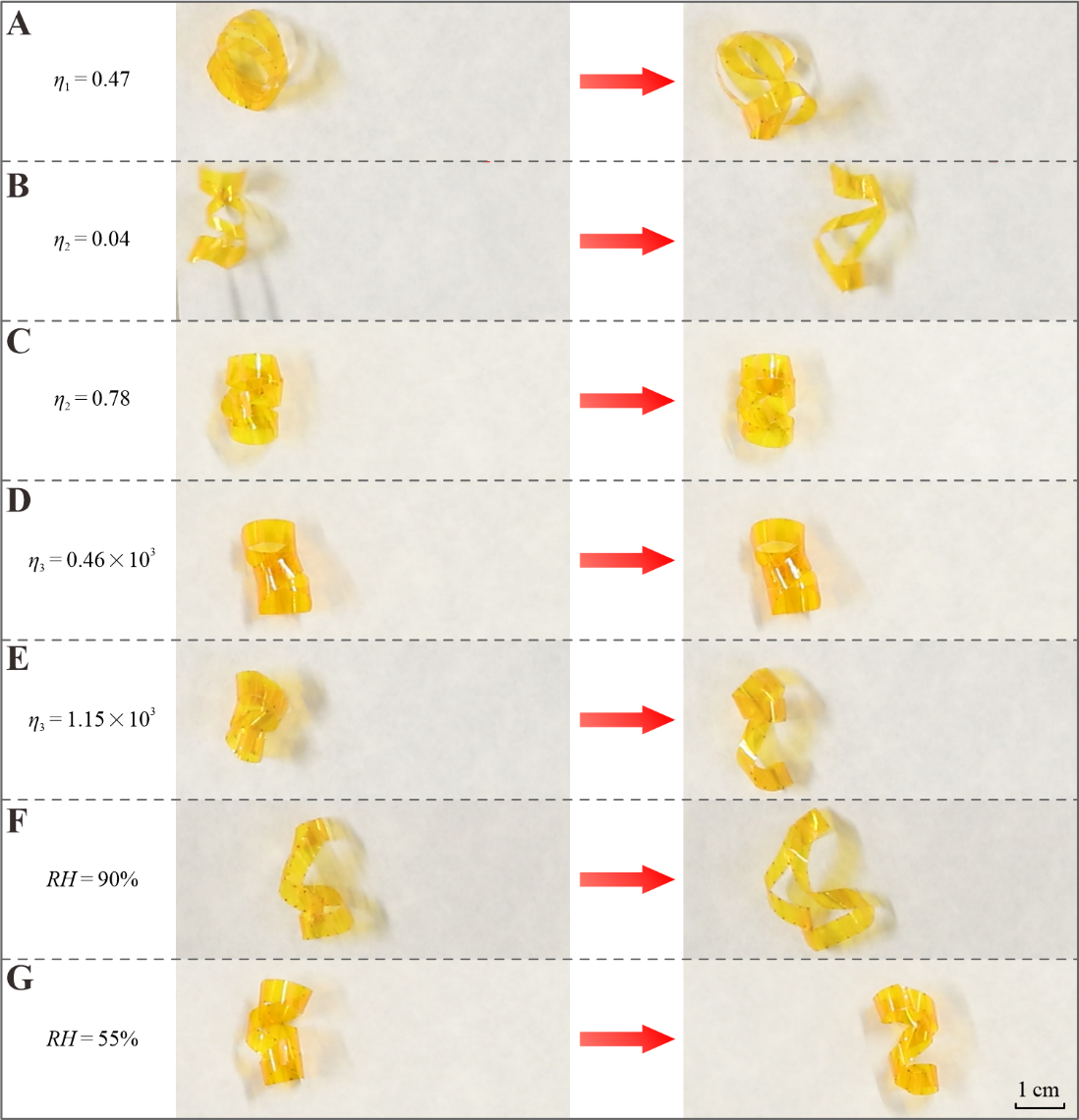


Figure S3. Demonstrations of the Dualicalbot with geometric or environmental parameters over the limiting ranges.


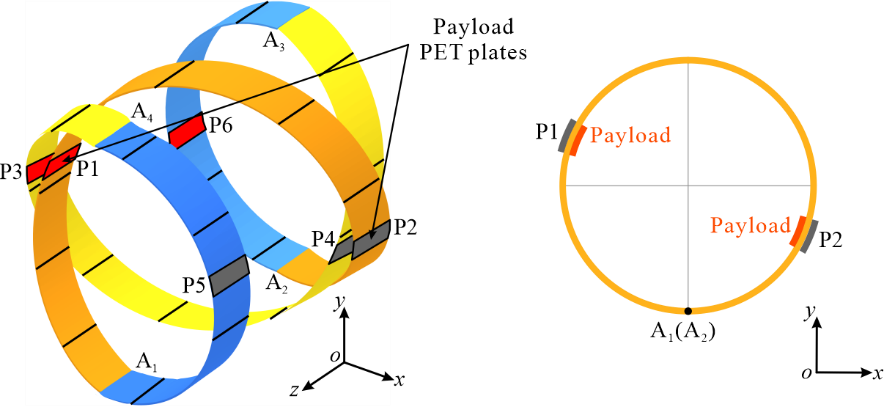


Figure S4. The locations of PET plates for payload.


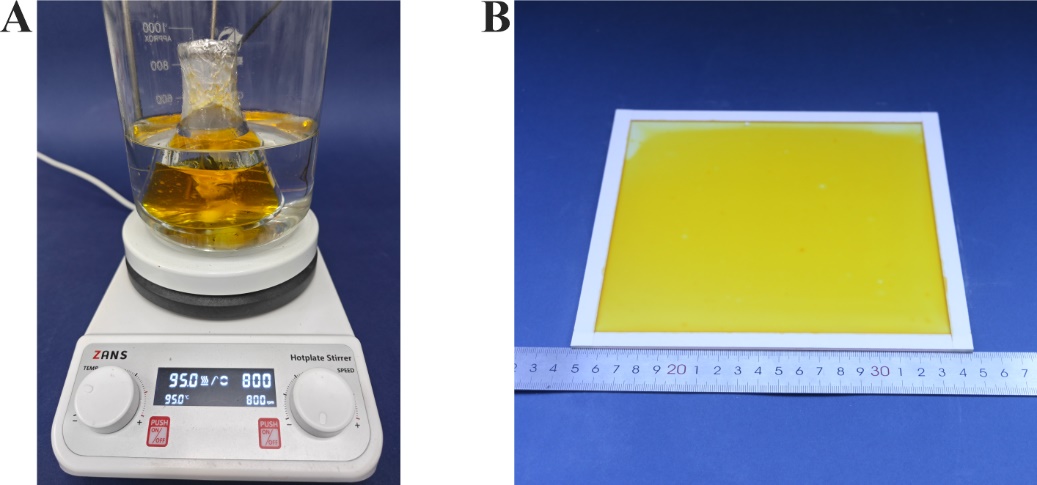


Figure S5. Preparation processes of the AG film. A) The solution mixed with DMF, AG powder, and phenol red powder is heated and stirred for 4.5 hours. B) The mixed solution is spread on the chromatographic plate with the size of 18 mm × 18 mm.


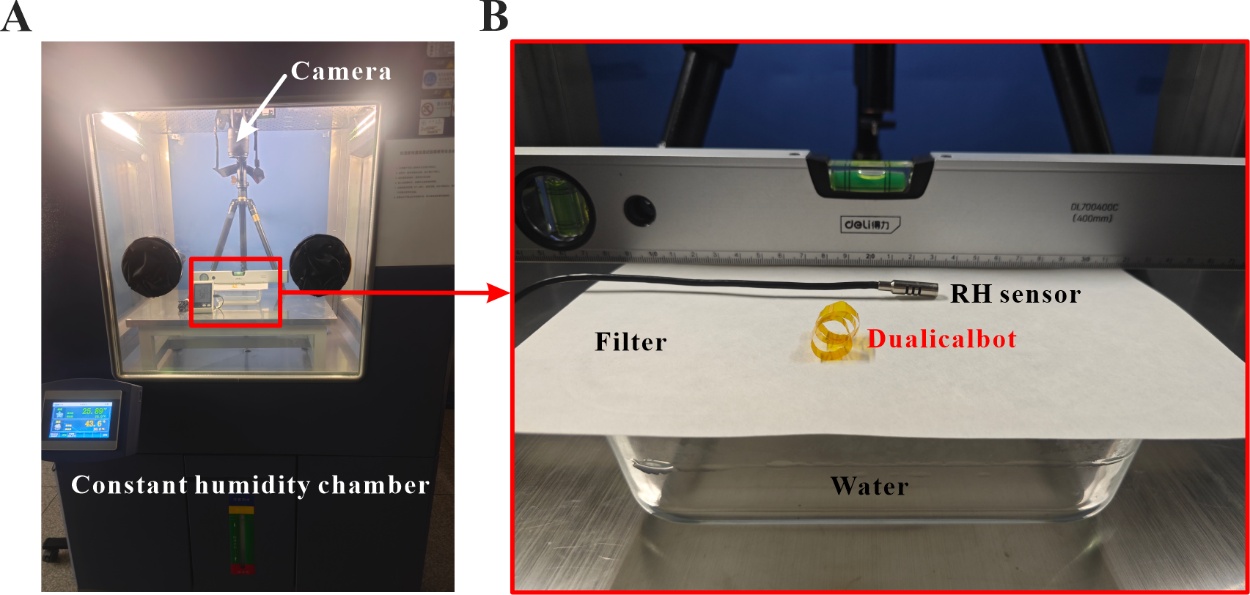


Figure S6. Setup of the rolling experiments of the Dualicalbot.


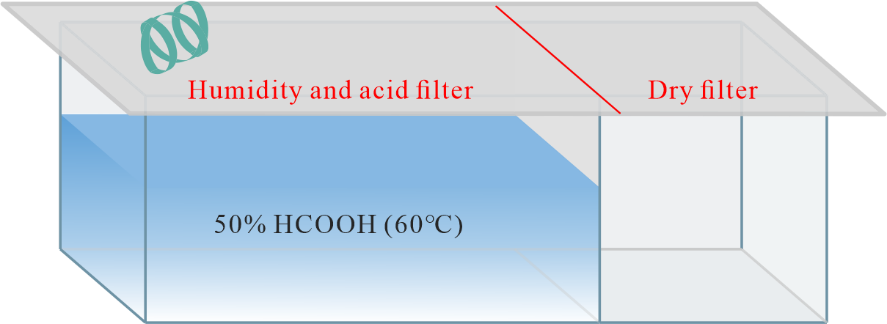


Figure S7. The experimental setup of the acid environmental detection robot.


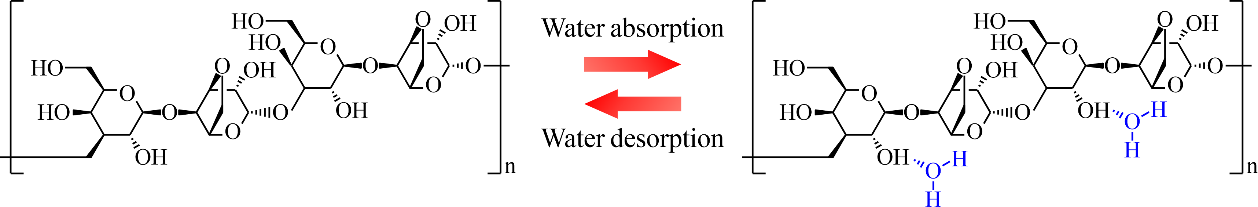


Figure S8. The chemical structure and humidity exchange mechanism of the AG film.


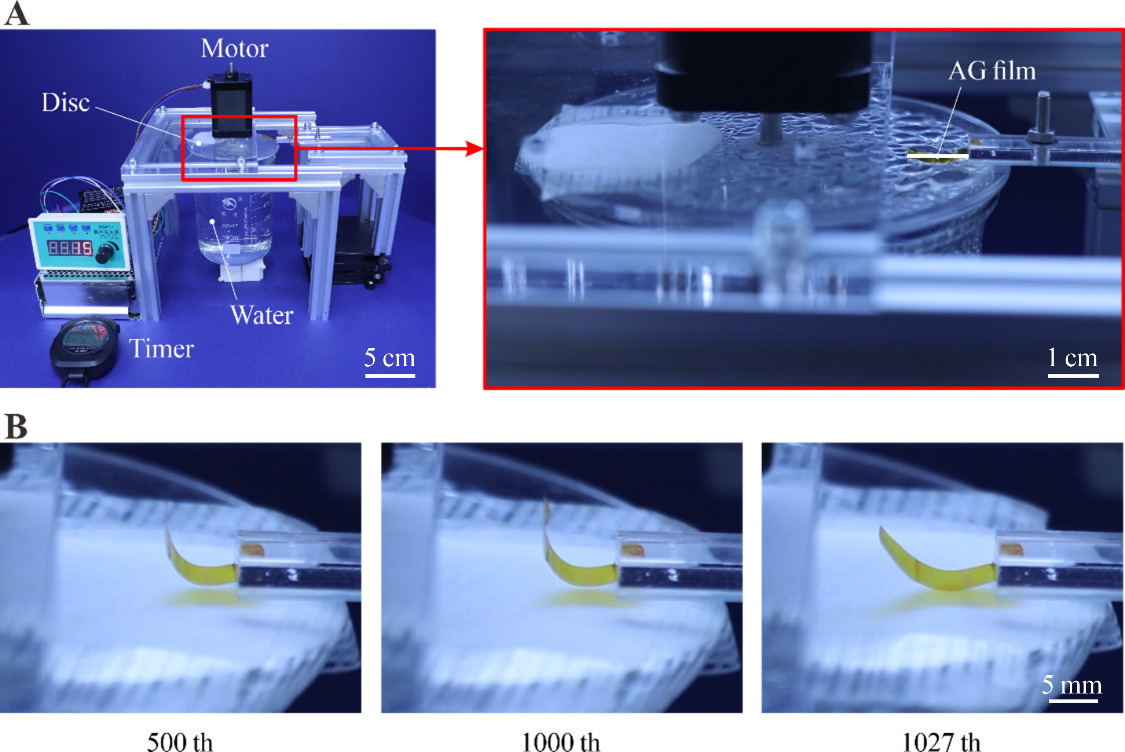


Figure S9. The fatigue life-span test of the AG film. A) Experimental setup. B) Experimental snapshot with the film thickness of 15 μm and the relative humidity of 90%.


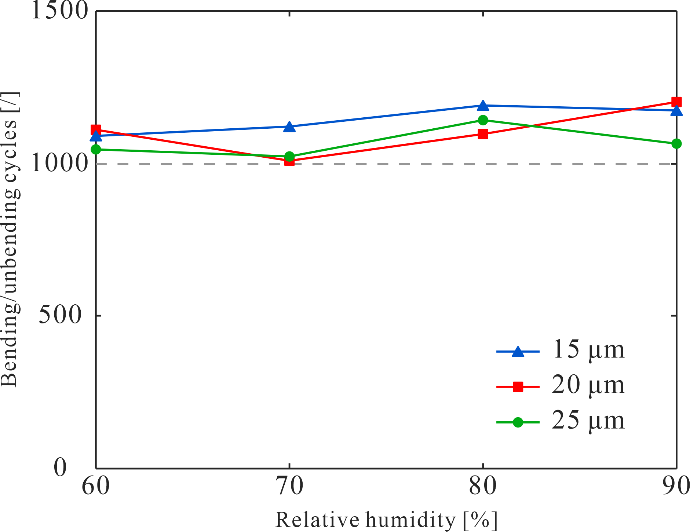


Figure S10. Experimental results of the fatigue life-span tests of the AG film.


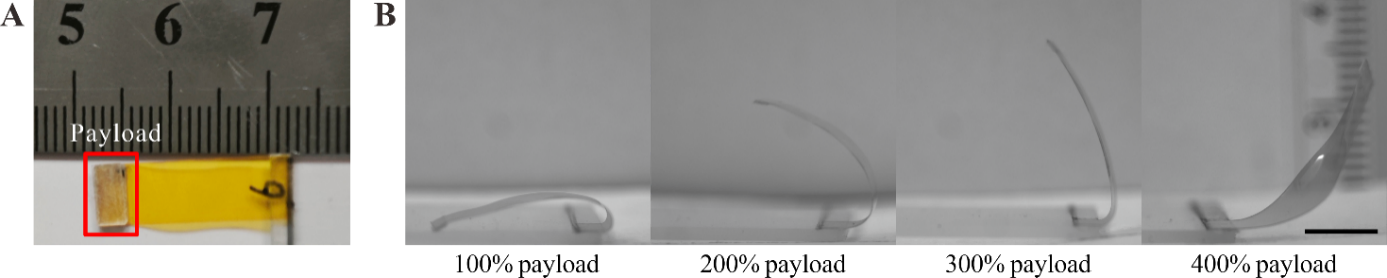


Figure S11. The payload capacity experiments of the AG film. A) PET strips are attached on the free end of the AG film as the payload. B) Experimental snapshots of the AG film with different payloads (scale bar: 5 mm).


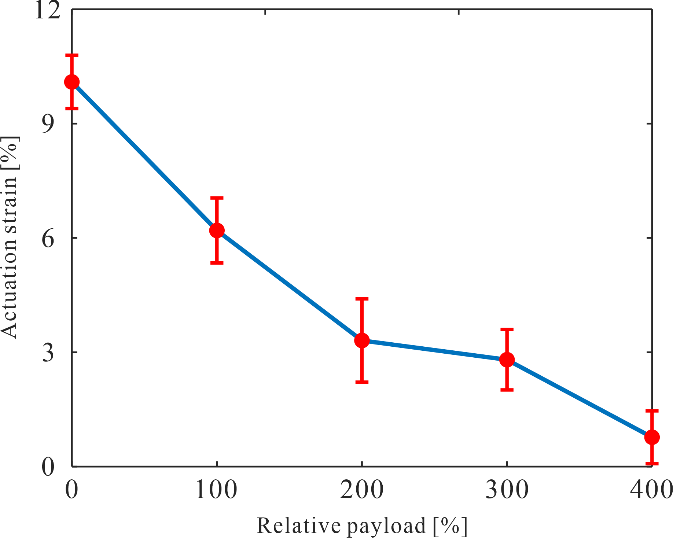


Figure S12. Experimental results of the payload capacity of the AG film. The red points are obtained from experimental data, which contain the error bars of at least three measurements.


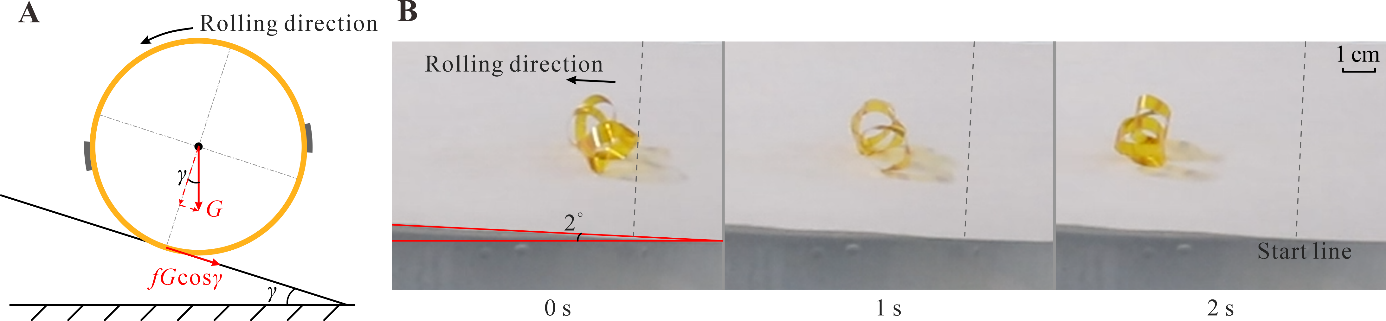


Figure S13. The analyses of the Dualicalbot rolling on a slope. A) Theoretical model. B) Snapshots of the experimental validation.

# Tables S1 to S5

Table S1. The water absorption and desorption rates of AG films with different thicknesses and different projected lengths in different relative humidity (the width of films is 6 mm).

| Thickness  (μm) | Projected length  (mm) | Relative humidity  (%) | Absorption rate  (×10^-8^ J/s) | Desorption rate  (×10^-8^ J/s) |
| --- | --- | --- | --- | --- |
| 10 | 18 | 60 | 13.58 | 1.04 |
| 15 | 18 | 60 | 16.85 | 1.27 |
| 20 | 18 | 60 | 18.76 | 1.46 |
| 25 | 18 | 60 | 22.28 | 1.64 |
| 30 | 18 | 60 | 22.37 | 1.79 |
| 20 | 16 | 60 | 16.58 | 1.22 |
| 20 | 18 | 60 | 19.14 | 1.46 |
| 20 | 20 | 60 | 22.99 | 1.72 |
| 20 | 22 | 60 | 25.72 | 1.98 |
| 20 | 24 | 60 | 28.86 | 2.25 |
| 10 | 18 | 40 | 1.82 | 0.20 |
| 10 | 18 | 45 | 3.32 | 0.30 |
| 10 | 18 | 50 | 5.00 | 0.46 |
| 10 | 18 | 55 | 8.72 | 0.69 |
| 10 | 18 | 60 | 13.59 | 1.04 |
| 10 | 18 | 65 | 22.50 | 1.56 |
| 10 | 18 | 70 | 36.74 | 2.35 |

Table S2. The relationship between the thickness of AG film and the solution spread on a plate.

| Thickness (μm) | Solution (mL) |
| --- | --- |
| 15 | 20.45 |
| 20 | 27.25 |
| 25 | 34.10 |

Table S3. The geometric parameters, limiting parameters and experimental rolling speed of the Dualicalbot in Figure 3D-3I.

| *a*  (mm) | *b*  (mm) | *γ*  (°) | *μ*  (μm) | $\eta_{1}$ | $\eta_{2}$ | $\eta_{3}$ | *v*  (BL s^-1^) |
| --- | --- | --- | --- | --- | --- | --- | --- |
| 15 | 5 | 60 | 15 | 0.428 | 0.250 | 0.637×10^3^ | 1.892 |
| 16 | 5 | 60 | 15 | 0.434 | 0.234 | 0.679×10^3^ | 1.944 |
| 17 | 5 | 60 | 15 | 0.439 | 0.220 | 0.722×10^3^ | 2.082 |
| 18 | 5 | 60 | 15 | 0.444 | 0.208 | 0.764×10^3^ | 2.240 |
| 19 | 5 | 60 | 15 | 0.449 | 0.197 | 0.807×10^3^ | 2.588 |
| 19 | 6 | 60 | 20 | 0.433 | 0.236 | 0.605×10^3^ | 1.911 |
| 20 | 6 | 60 | 20 | 0.437 | 0.225 | 0.637×10^3^ | 2.045 |
| 21 | 6 | 60 | 20 | 0.442 | 0.214 | 0.669×10^3^ | 2.203 |
| 22 | 6 | 60 | 20 | 0.446 | 0.204 | 0.701×10^3^ | 2.335 |
| 23 | 6 | 60 | 20 | 0.450 | 0.195 | 0.732×10^3^ | 2.555 |
| 23 | 7 | 60 | 25 | 0.436 | 0.228 | 0.586×10^3^ | 2.083 |
| 24 | 7 | 60 | 25 | 0.440 | 0.218 | 0.611×10^3^ | 2.208 |
| 25 | 7 | 60 | 25 | 0.444 | 0.210 | 0.637×10^3^ | 2.303 |
| 26 | 7 | 60 | 25 | 0.447 | 0.202 | 0.662×10^3^ | 2.400 |
| 27 | 7 | 60 | 25 | 0.450 | 0.194 | 0.688×10^3^ | 2.567 |
| 17 | 4 | 60 | 15 | 0.450 | 0.176 | 0.722×10^3^ | 2.093 |
| 17 | 5 | 60 | 15 | 0.439 | 0.220 | 0.722×10^3^ | 2.082 |
| 17 | 6 | 60 | 15 | 0.422 | 0.264 | 0.722×10^3^ | 2.134 |
| 17 | 7 | 60 | 15 | 0.406 | 0.308 | 0.722×10^3^ | 2.099 |
| 18 | 6 | 53 | 15 | 0.346 | 0.176 | 0.764×10^3^ | 2.242 |
| 18 | 6 | 56 | 15 | 0.378 | 0.205 | 0.764×10^3^ | 2.199 |
| 18 | 6 | 60 | 15 | 0.428 | 0.250 | 0.764×10^3^ | 2.209 |
| 18 | 6 | 63 | 15 | 0.440 | 0.291 | 0.764×10^3^ | 2.247 |

Table S4. The tape parameters, relative humidity, payload mass, and experimental rolling speed of the Dualicalbot in Figure 3J-3L and Figure 4B, where *m*_p_ and *m*_0_ are the mass of payload and that of the robot.

| *c*  (mm) | *e*  (/) | $m_{p}/m_{0}$ (%) | *RH*  (%) | *v*  (BL s^-1^) |
| --- | --- | --- | --- | --- |
| 1.5 | *a*/2 | 0 | 70 | 2.648 |
| 2.0 | *a*/2 | 0 | 70 | 2.486 |
| 2.5 | *a*/2 | 0 | 70 | 2.400 |
| 3.0 | *a*/2 | 0 | 70 | 2.113 |
| 1.5 | *2a*/3 | 0 | 70 | 2.451 |
| 1.5 | *5a*/6 | 0 | 70 | 2.211 |
| 1.5 | *a*/2 | 0 | 60 | 0.820 |
| 1.5 | *a*/2 | 0 | 65 | 1.456 |
| 1.5 | *a*/2 | 0 | 70 | 2.209 |
| 1.5 | *a*/2 | 0 | 75 | 3.454 |
| 1.5 | *a*/2 | 0 | 80 | 4.744 |
| 1.5 | *a*/2 | 0 | 85 | 5.832 |
| 1.5 | *a*/2 | 50 | 85 | 4.932 |
| 1.5 | *a*/2 | 100 | 85 | 3.444 |

Table S5. Locomotion metrics of some typical untethered soft robots shown in Figure 4A.

| Drive method | Drive medium | Body length (mm) | Speed (BL s^-1^) | Ref. |
| --- | --- | --- | --- | --- |
| Constant environment | Humidity | 12.1 | 5.8 | *This work* |
|  | Humidity | 18 | 0.714 | *34* |
|  | Humidity | 30 | 0.0038 | *35* |
|  | Light | 25 | 0.1 | *26* |
|  | Thermo | 3 | 1 | *24* |
|  | Thermo | 5 | 0.15 | *19* |
|  | Thermo | 40 | 0.13 | *23* |
|  | Thermo | 60 | 0.05 | *20* |
|  | Thermo | 35 | 0.04 | *22* |
| Modulated  environment | Humidity | 30 | 0.24 | *32* |
|  | Humidity | 20 | 0.084 | *33* |
|  | Humidity | 27 | 0.035 | *36* |
|  | Humidity | 16 | 0.024 | *32* |
|  | Light | 1 | 21.63 | *30* |
|  | Light | 30 | 3.47 | *41* |
|  | Light | 20 | 2 | *27* |
|  | Light | 10 | 0.67 | *41* |
|  | Light | 0.75 | 0.2 | *28* |
|  | Light | 20 | 0.05 | *42* |
|  | Light | 16 | 0.023 | *25* |
|  | Light | 10 | 0.019 | *29* |
|  | Thermo | 90 | 0.01 | *21* |
|  | Magnet | 20 | 1 | *9* |
|  | Magnet | 30 | 0.83 | *12* |
|  | Magnet | 45 | 0.78 | *8* |
|  | Magnet | 12.4 | 0.2 | *10* |
|  | Magnet | 15.6 | 0.13 | *10* |
|  | Magnet | 10 | 0.12 | *11* |
|  | Magnet | 0.03 | 3 | *7* |
|  | Magnet | 27 | 0.037 | *43* |
|  | Electron | 20 | 2.1 | *6* |
|  | Electron | 9 | 1.2 | *6* |
|  | Electron | 20 | 1 | *4* |
|  | Electron | 30 | 0.01 | *5* |

# Legends for Movies S1 to S5

Movie S1.

Fabrication and motion process of the Dualicalbot.

Movie S2.

Rolling demonstration of the Dualicalbot.

Movie S3.

Rolling demonstration of the Dualicalbot with optimal geometric parameters.

Movie S4.

Demonstrations of the Dualicalbot with different payload.

Movie S5.

Demonstration of the acid environment detection robot.

# References

[S1] L. Zhang, H. Liang, J. Jacob, P. Naumov, *Nat. Commun.* **2015**, 1, 7429.

[S2] G. Tang, X. Zhao, S. Liu, D. Mei, C. Zhao, L. Li, Y. Wang, L. Sun, Q. Zhao, L. Che, M. Li, Xu Leng, Y. Long, Y. Lu, *Adv. Funct. Mater.* **2024**, 2412254.

[S3] L. Fu, W. Zhao, J. Ma, M. Yang, X. Liu, L. Zhang, Y. Chen, *Research* **2022**, 9832901.
